# Expression and function of pannexins in the inner ear and hearing

**DOI:** 10.1186/s12860-016-0095-7

**Published:** 2016-05-24

**Authors:** Hong-Bo Zhao

**Affiliations:** Department of Otolaryngology, University of Kentucky Medical Center, 800 Rose Street, Lexington, KY 40536 USA

**Keywords:** Pannexin, ATP release, Endocochlear potential, Active cochlear amplification, Cell degeneration, Hearing loss, Inner ear

## Abstract

Pannexin (Panx) is a gene family encoding gap junction proteins in vertebrates. So far, three isoforms (Panx1, 2 and 3) have been identified. All of three Panx isoforms express in the cochlea with distinct expression patterns. Panx1 expresses in the cochlea extensively, including the spiral limbus, the organ of Corti, and the cochlear lateral wall, whereas Panx2 and Panx3 restrict to the basal cells of the stria vascularis in the lateral wall and the cochlear bony structure, respectively. However, there is no pannexin expression in auditory sensory hair cells. Recent studies demonstrated that like connexin gap junction gene, Panx1 deficiency causes hearing loss. Panx1 channels dominate ATP release in the cochlea. Deletion of Panx1 abolishes ATP release in the cochlea and reduces endocochlear potential (EP), auditory receptor current/potential, and active cochlear amplification. Panx1 deficiency in the cochlea also activates caspase-3 cell apoptotic pathway leading to cell degeneration. These new findings suggest that pannexins have a critical role in the cochlea in regard to hearing. However, detailed information about pannexin function in the cochlea and Panx mutation induced hearing loss still remain largely undetermined. Further studies are required.

## Background

Gap junction is an intercellular channel and exists in both vertebrates and invertebrates. However, the gap junctional proteins in vertebrates and invertebrates are encoded by different genes. In vertebrates, gap junctional proteins are encoded by a connexin gene family, whereas in invertebrates they are encoded by an unrelated innexin gene family. About 15 years ago, by application of genoinformatics, an innexin homologue, termed pannexin, was found in the human genome [1–3]. Later, Panx expression in rodents, zebrafish, and an invertebrate chordate was also identified [3].

### Pannexin genomics and expression

So far, three pannexin isoforms (Panx1, 2, and 3) have been cloned from the human and mouse genomes [2, 3]. Panx1 gene is located on human chromosome 11q14.3 in a 700 kb interval between the genes *CRSP6* and *MRE11*, Panx2 on human chromosome 22q13.31–q13.33, and Panx3 on human chromosome 11q24.2 in a 150 kb interval between the telomeric border of the cluster of olfactory gene family 8 and *TBRG1*. In the mouse, Panx1, 2, and 3 genes are located on chromosome 9, 15, and 9, respectively [3]. Phylogenetic analysis demonstrates that pannexin is highly conserved in Nematoda, Mollusca, Arthropoda and mammals [1, 3], implying that pannexins may have important functions.

### Pannexin channels and functions

Despite the lack of similar sequences with connexins, pannexin proteins share large similarities at the structural and functional levels. Pannexin proteins also possess four transmembrane domains, two extracellular loops, one intracellular loop, and intracellular N- and C-termini [3]. The profile of pannexin channel permeability is similar to that of connexin channels, permeable to ions and small molecules up to 1 kDa [4]. However, despite general parallels with connexin channels, the properties and pharmacology of pannexin channels are distinct [5, 6]. First, it is becoming apparent that unlike connexins to form integrated gap junction channels, pannexins mainly function as plasma membrane channels ('hemichannels') on the cell surface to provide an intracellular-extracellular conduit [7–10]. Second, Panx1 channels demonstrate larger currents with increasing depolarization, faster kinetics of pore opening, larger unitary conductance (~500 pS, compared to a maximum ~ 300 pS in Cx43 hemichannels), very weak voltage gating, and multiple substates in single-channel recordings [2, 11, 12]. Third, both homomeric and heteromeric (Panx1/Panx2) channels show significantly higher sensitivity to carbenoxolone and probenecid [13, 14]. Finally, in contrast to the connexin channels, which are highly sensitive to Ca^++^ and can be closed by the physiological levels of extracellular Ca^++^ (1–2 mM), pannexin channels are insensitive to Ca^++^ and can open and function at the physiological extracellular levels of Ca^++^ [11]. These specific properties of the channel activity imply that pannexin channels can play an important role in a wider range of physiological function and pathological processes. So far, pannexins have been found to play important functions in the ATP release, Ca^++^ wave propagation, vasodilation, ischemic cell death, inflammatory response, and release of synaptic neurotransmitters [15–17]. This review mainly focuses on the expression and function of pannexins in the inner ear and in hearing.

### Pannexin expression in the cochlea

**Fig. 1:**
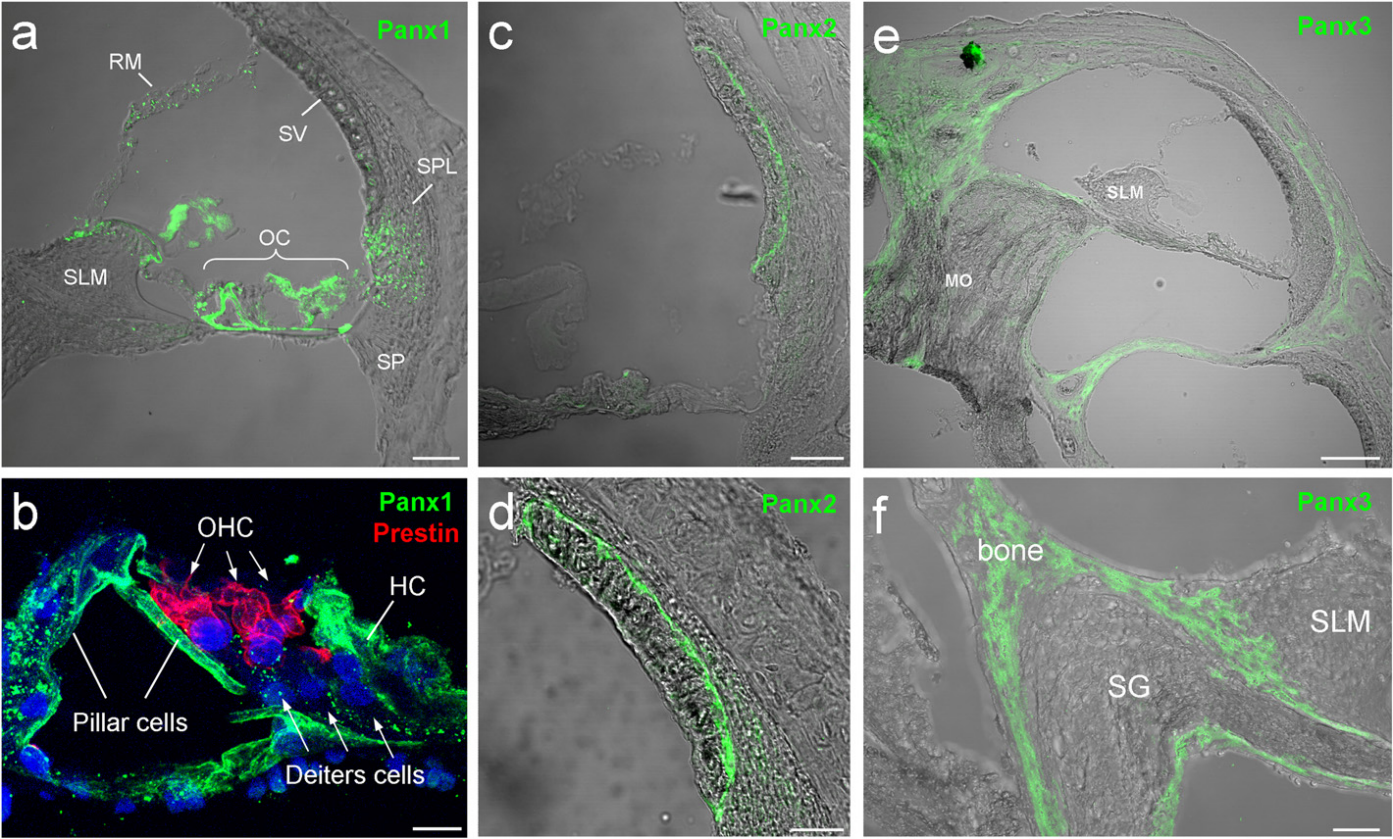
Immunofluorescent labeling for Panx1, 2, and 3 in the cochlea. **a-b**: Immunofluorescent staining for Panx1. Outer hair cells (OHCs) are visualized by prestin staining (red) in (panel **b**). **c-d**: Immunofluorescent staining for Panx2. **e-f**: Immunofluorescent staining for Panx3. HC: Hensen cell; MO: modiolus; OC: organ of Corti; RM: Reissner’s membrane; SG: spiral ganglion; SLM: spiral limbus; SP: spiral prominence; SPL: spiral ligament; SV: stria vascularis. Scale bar: 50 μm in (**a**, **c**), 100 μm in E, 10 μm in (**b**, **d** and **f**). Modified from [18]

Like connexins, pannexins have ubiquitous expression. In the mammalian cochlea, we found that all three pannexin isoforms have expressions [18]. Panx1 expresses at the cochlear supporting cells, the spiral limbus, and the cochlear lateral wall. Panx2 only expresses at the basal cell layer in the stria vascularis in the cochlear lateral wall, and Panx3 expression is restricted to the cochlear bony structure (Fig. 1). However, like connexins, the auditory sensory hair cells have no pannexin expression (Fig. 1, also see [18]). These distinct expression patterns strongly suggest that pannexins have important functions in the inner ear and in hearing.

### Pannexin deficiency induced hearing loss

**Fig. 2:**
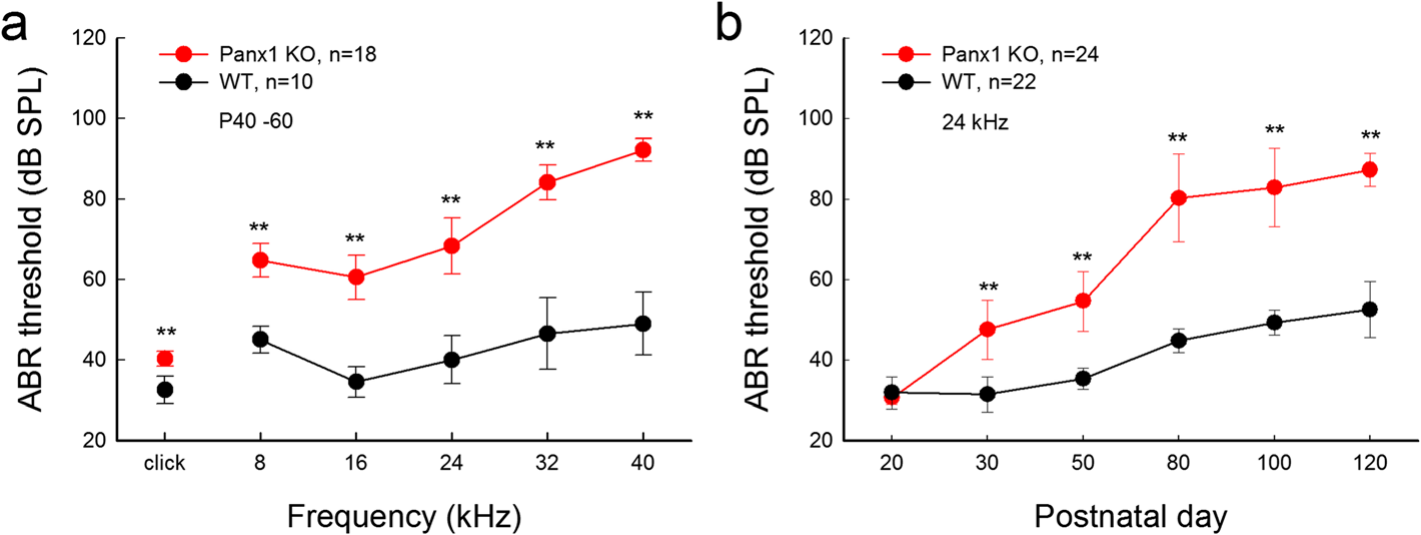
Panx1 deletion induced hearing loss. **a**: Hearing loss as measured by ABR thresholds, which are significantly increased in Panx1 KO mice. The increase is large at high-frequency range. **b**: Hearing loss is progressive. **P < 0.001, two-way ANOVA with a Bonferroni correction. Modified from [20, 21]

It has been well-known that connexin mutations can cause hearing loss [19]. Our recent studies showed that Panx1 deficiency also induces hearing loss [20, 21]. We found that the Panx1 deficient mice have hearing loss; the hearing loss appeared progressive, moderate to severe, and severe at high-frequency range (Fig. 2, also see [20, 21]). Although pannexin mutation induced hearing loss has not been identified yet, our findings strongly suggest that pannexin mutations also can induce hearing loss in humans.

### Pannexin function in the cochlea

#### Panx1 channels dominate ATP release in the cochlea

A major function of Panx1 channels is to release ATP [17]. ATP is an important energy source in cells and also acts as an important cell signaling molecule in the extracellular space, when it is released to the outside of cells. As mentioned above, pannexin channels possess relatively large pore size and are permeable to ATP. Due to Panx1 channels can work at normal physiological levels of Ca^++^, Panx1 channels in many organs and tissues act as a major conduit for ATP release under physiological conditions [22–25].

**Fig. 3:**
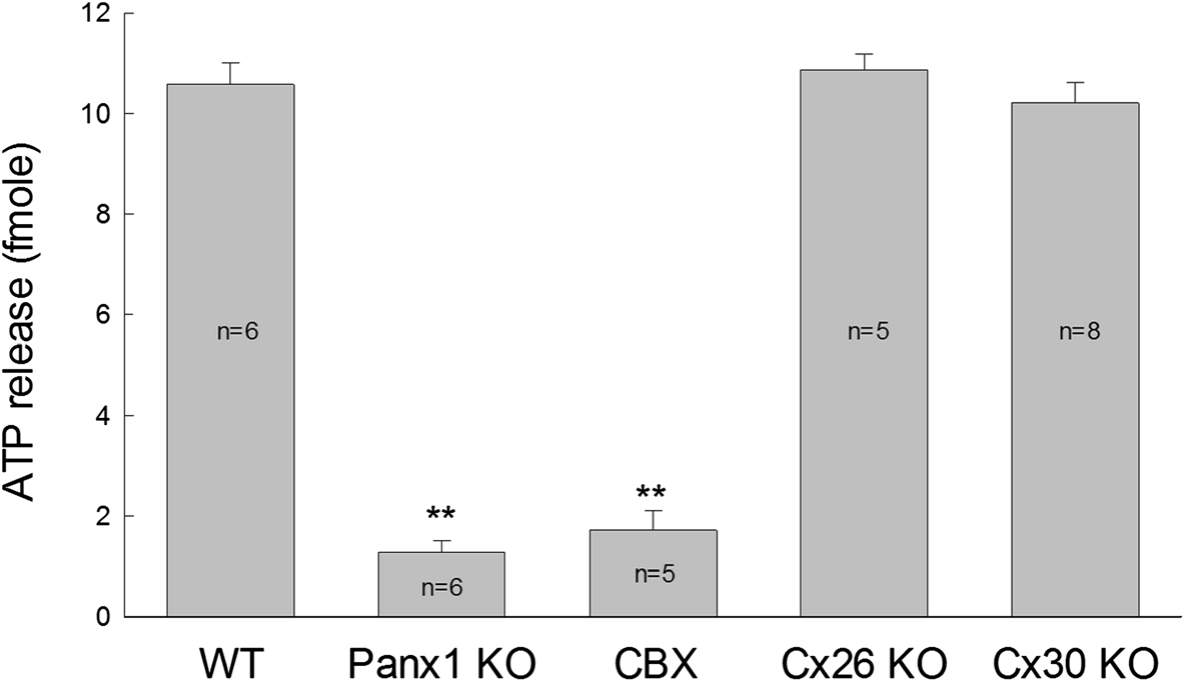
Panx1 channels dominate ATP release in the cochlea. Deletion of Panx1 and application of 0.1 mM carbenoxolone (CBX) but not deletion of Cx26 and Cx30, which are predominant connexin isoforms in the cochlea, eliminate ATP release in the cochlea. ATP release was measured in the normal extracellular solution which contains 2 mM Ca^++^. **P < 0.001, one-way ANOVA with a Bonferroni correction. Modified from [21]

In the cochlea, ATP physiologically exists in the endolymph and perilymph [26]. We previously found that gap junctional hemichannels are responsible for ATP release in the cochlea [27]. However, it was unclear at that time which hemichannels were responsible for ATP release. Recently, we found that deletion of Panx1 abolished ATP release in the cochlea, whereas deletion of Cx26 and Cx30, which are predominant connexin isoforms in the cochlea [28, 29], had little effect on ATP release under physiological conditions (Fig. 3, also see [21]). Moreover, it has been found that a gap junction channel antagonist carbenoxolone (CBX) could eliminate ATP release in the cochlea at the physiological level of extracellular Ca^++^ (2 mM) (Fig. 3, also see [21]). These new data demonstrate that ATP in the cochlea is mainly released via Panx1 channels under physiological conditions.

These new findings, however, are inconsistent with a previous report that Paxn1 “knockout” had no effect on ATP release in the cochlea [30]. In that study, ATP release was recorded by use of ATP sensory electrodes, which sensitivity is only at the micromolar levels and is too low to measure fmole to submicromolar levels of ATP in the cochlea [21, 26, 27]. Moreover, their used “Panx1 KO” mice were created by Cre-loxP technique. There is no information available for which Cre mouse line was used. Also, there were no experiments to check Panx1 expression in the cochlea. So, whether Panx1 was really deleted or not in the cochlea in that Panx1 ‘KO’ mouse remains unclear. In our experiments [21], we used a bioluminescent-based method to measure ATP release, which is the most sensitive and reliable method for measuring ATP release. We also used immunofluorescent staining and confirmed that Panx1 was deleted in the cochlea [20, 21]. Finally, we found that Cx26 and Cx30 deletion had little effect on ATP release in the cochlea under physiological conditions (Fig. 3, also see [21]). Thus, these new experiments and data provide strong evidence that Panx1 channels in the cochlea are responsible for ATP release under physiological conditions.

#### Ensuring of endocochlear potential and auditory receptor current/potential generation in the cochlea

Positive endocochlear potential (EP, +100 ~ 110 mV) in the cochlea is indispensable for hearing and is a driving force that compels K^+^ ions in the endolymph through the transduction channels at stereocilia of hair cells to produce auditory receptor current and potential, thereby initiating hearing. Positive EP is generated in the cochlear lateral wall. ATP is required for EP generation [31]. We found that deletion of Panx1 in the cochlear lateral wall reduced ATP release and EP generation, thereby reducing auditory receptor potential and causing hearing loss [21]. However, the detailed mechanisms for Panx1 channel ATP release and EP generation still remain unclear and require further studies in future.

#### The role of Panx1 in active cochlear amplification

We also found that deletion of Panx1 expression in the cochlea could reduce active cochlear amplification [20]. Distortion product otoacoustic emission (DPOAE) was reduced. Consistent with hearing loss, the reduction was severe at high frequencies [20, 21]. DPOAE in mammals is mainly produced by outer hair cell electromotility based active cochlear amplification. However, as mentioned above (Fig. 1b, also see [18]), outer hair cells have no any pannexin expression. Currently, the detailed mechanisms for how Panx1 deficiency can affect outer hair cell electromotility and reduce active cochlear amplification remain unclear. It could be a consequence from EP and auditory receptor current/potential reduction due to Panx1 knockout reduced ATP release (Fig. 3 and Fig. 7 in [21]).

#### Other downstream effects of ATP release reduction due to Panx1 deficiency in the cochlea

Extracellular ATP is an important extracellular cell signaling molecule and can activate purinergic P2 receptors to play broad roles in many physiological functions and pathological processes [32, 33]. In the cochlea, ATP can elevate intracellular Ca^++^ concentration in hair cells modifying sound transduction and neurotransmission [34], mediate hearing sensitivity and extent the dynamic range of hearing [35–37], and synchronize auditory nerve activity during development [38, 39]. In addition, ATP can also activate purinergic P2X receptors to mediate stimulation of parasensory cation absorption in the cochlea [40]. We also found that ATP can activate P2X receptors to mediate outer hair cell electromotility [27, 41], gap junctional coupling [42], K^+^-sinking [43], and EP generation [21]. Recently, it has been found that mutations of P2X2 purinergic receptors induce autosomal dominant nonsyndromic hearing loss DFNA41 [44, 45] and increase susceptibility to noise stress [44], indicating that Panx1-ATP-P2X receptor-mediated purinergic cell signaling has a critical role in hearing. Thus, Panx1 deficiency leading to ATP release reduction may have more broad effects on the cochlear function and hearing.

### The role of Panx1 in cell degeneration in the cochlea

One important function of pannexin channels is to participate in cell apoptotic process. It has been reported that the activation of Caspase-3 cell apoptotic pathway can permanently open Panx1 channels leading to cell apoptosis and death [46–50]. Recently, we found that Panx1 deletion also activates Caspase-3 apoptotic pathway in the cochlea leading to cell degeneration [20]. The activity of caspase-3 was detectable in both hair cells and cochlear supporting cells in Panx1 knockout (KO) mice. However, hair cells have neither connexin nor pannexin expression [18, 29]. How Panx1 deletion causes hair cell degeneration currently remains unknown.

## Conclusions

Like connexins, pannexins also have extensive expression in the cochlea [18]. Panx1 is a predominant isoform. Our recent studies showed that Panx1 deficiency causes hearing loss, abolishes ATP release in the cochlea, and reduces EP and auditory receptor potential [20, 21]. Panx1 deficiency also activates caspase-3 cell apoptotic pathway in the cochlea leading to cell degeneration [20]. However, pannexin function in the cochlea and in hearing still remains largely undetermined. Connexin mutations can induce a high incidence of hearing loss, responsible for >50% of nonsyndromic deafness [19]. These new findings strongly suggest that Panx1 mutations may also be able to induce hearing loss in humans, which requires further study in future.
